# Update on the Role of High-Flow Nasal Cannula in Infants with Bronchiolitis

**DOI:** 10.3390/children8020066

**Published:** 2021-01-20

**Authors:** Valentina Fainardi, Lara Abelli, Maria Muscarà, Giovanna Pisi, Nicola Principi, Susanna Esposito

**Affiliations:** 1 Pediatric Clinic, Pietro Barilla Children’s Hospital, Department of Medicine and Surgery, University of Parma, 43126 Parma, Italy; valentina.fainardi@gmail.com (V.F.); abellilara@gmail.com (L.A.); mariamus87@gmail.com (M.M.); gpisi@ao.pr.it (G.P.); 2 Università degli Studi di Milano, 20122 Milan, Italy; nicola.principi@unimi.it

**Keywords:** bronchiolitis, CPAP, BiPAP, HFNC, oxygen therapy

## Abstract

Bronchiolitis (BR), a lower respiratory tract infection mainly caused by respiratory syncytial virus (RSV), can be very severe. Presently, adequate nutritional support and oxygen therapy remain the only interventions recommended to treat patients with BR. For years, mild BR cases were treated with noninvasive standard oxygen therapy (SOT), i.e., with cold and poorly or totally non-humidified oxygen delivered by an ambient headbox or low-flow nasal cannula. Children with severe disease were intubated and treated with invasive mechanical ventilation (IMV). To improve SOT and overcome the disadvantages of IMV, new measures of noninvasive and more efficient oxygen administration have been studied. Bi-level positive air way pressure (BiPAP), continuous positive airway pressure (CPAP), and high-flow nasal cannula (HFNC) are among them. For its simplicity, good tolerability and safety, and the good results reported in clinical studies, HFNC has become increasingly popular and is now widely used. However, consistent guidelines for initiation and discontinuation of HFNC are lacking. In this narrative review, the role of HFNC to treat infants with BR is discussed. An analysis of the literature showed that, despite its widespread use, the role of HFNC in preventing respiratory failure in children with BR is not precisely defined. It is not established whether it can offer greater benefits compared to SOT and when and in which infants it can replace CPAP or BiPAP. The analysis of the results clearly indicates the need for multicenter studies and official guidelines. In the meantime, HFNC can be considered a safe and effective method to treat children with mild to moderate BR who do not respond to SOT.

## 1. Background

Bronchiolitis (BR), a lower respiratory tract infection mainly caused by respiratory syncytial virus (RSV), can be very severe [1]. Hospitalization may be required in up to 10% of cases [2,3], with up to 23.8% of these patients needing critical care for respiratory impairment or apnea [4,5]. Although BR can be diagnosed in adults and elderly people, it is infants, particularly the youngest, that are the subjects more affected by the disease. Those born prematurely or those suffering from coexisting heart disease, chronic respiratory illness, neuromuscular disease, or immunodeficiencies are the subjects with the highest risk of severe forms of BR [1]. Younger infants have peculiar anatomical and developmental characteristics that favor respiratory failure development when infected by RSV or other respiratory viruses. In the first months of life, lung alveoli are still developing in both number and function [6], respiratory muscles are not totally effective [7], and the caliber of bronchioles is very small [1]. Partial or total occlusion of bronchioles by intraluminal mucus and debris accumulation leading to air trapping or atelectasis occurs more frequently in young children than in older subjects and adults [1]. Increased airway resistance is associated with increased breathing workload and severe hypercapnia and hypoxemia, which can cause relevant immediate and long-term clinical problems to develop [8]. The risk of an adverse outcome is further aggravated by the evidence, as confirmed by the high rate of apnea episodes, that respiratory virus infections in younger infants can be associated with profound central autonomic dysfunctions leading to prolonged pauses of breathing [8].

To prevent respiratory failure in BR, several medical therapies have been suggested. However, none of them, including corticosteroids and bronchodilators, have been found highly effective as stated by official USA and UK guidelines [9,10]. Only the Italian recommendations suggest a trial with nebulized salbutamol, especially in children with familiar or personal history for asthma, allergy, or atopy [11]. In the case of no benefit in air entry and/or respiratory distress within 15–30 min, the drug must be withdrawn and no more prescribed. Administration of hypertonic saline is debated, as study results have been conflicting and studies with positive data were frequently carried out in children with mild disease or without factors favoring the evolution of the disease in severe forms of BR [12].

Presently, adequate nutritional support and oxygen therapy remain the only interventions recommended to treat patients with BR [1], although details for oxygen administration and suspension can slightly vary among experts [9,10]. For years, mild BR cases were treated with noninvasive standard oxygen therapy (SOT), i.e., with cold and poorly or totally non-humidified oxygen delivered by ambient headbox or low-flow nasal cannula [11,12]. Children with severe disease were intubated and treated with invasive mechanical ventilation (IMV). However, both these methods were considered not completely satisfactory. With SOT, risk of leaking air around the oxygen source was high. The oxygen concentration was not controllable and could significantly vary during administration. Moreover, only flows of 2 L/min were allowed as higher flows of cold and not or poorly humidified oxygen could cause drying of the mucosa, increase in the inflammatory state and airway resistance, and impaired mucociliary function [13]. Hypoxemia could only be partially reduced, respiratory fatigue was poorly modified, and thick intraluminal mucus was not loosened. On the other hand, IMV has several limitations. It is expensive and exposes the patient to potentially toxic sedative medications. Moreover, it can lead to skin or eye complications, gastric distension with feeding problems, airway and tracheal injury, air leak syndromes, bronchopulmonary dysplasia, hemodynamic compromise, and neurologic injury. Finally, risk of superimposed infection is not negligible [14].

To improve SOT and overcome the above-mentioned disadvantages of IMV, new measures of noninvasive, more efficient oxygen administration have been studied. Bi-level positive airway pressure (BiPAP), continuous positive airway pressure (CPAP), and high-flow nasal cannula (HFNC) are among them [15,16]. For its simplicity, good tolerability and safety, and the good results reported in the first clinical studies, HFNC has become increasingly popular and is now widely used. However, not all the problems related to HFNC use are presently solved. Guidelines for initiation and discontinuation of HFNC are lacking. The efficacy of HFNC compared to SOT and other recently developed noninvasive methods is not precisely defined. In this narrative review, the role of HFNC for treatment of infants with BR is discussed. To collect information, we searched for publications and abstracts in PubMed and Embase, and for systematic reviews in the Cochrane database from January 2010 to December 2020. Search strategies as bronchiolitis OR bronchopneumonia OR respiratory syncytial virus OR respiratory syncytial viruses OR RSV AND HFNC OR high flow nasal cannula OR humidified high-flow nasal cannula OR HFNC OR heated humidified high-flow nasal cannula OR high flow oxygen OR nasal high flow were used. A total of 123 papers were selected.

## 2. High-Flow Nasal Cannula (HFNC) Systems

### 2.1. The Device

The HFNC system consists of a flow generator (air/oxygen blender, turbine or Venturi mask) that provides a flow of gas containing oxygen from 21 to 100% up to 60 L/min. The gas is heated and humidified through an active heated humidifier and delivered through a heated tube. The circuit is connected to a silicone nasal cannula of different sizes to fit the patient’s nostrils. The flow of gas, fraction of inspired oxygen (FiO2), and temperature can be independently regulated according to the patient’s needs and characteristics. The size of cannulas should be varied according to age and body weight of the patient, paying attention so that their outer diameter is no more than two-thirds that of the nares because of the risk of unexpected elevations in airway pressure and the following risk of air leak [17]. Originally restricted to intensive care units, use of HFNC has now expanded to emergency departments, inpatient pediatric wards, pre- and interhospital transport settings and, although rarely, to home treatment. The availability of very simple and portable devices has greatly favored its diffusion as a substitute for BiPAP or CPAP, despite the US Food and Drug Administration [18] only approving HFNC as optimal humidification of oxygen therapy and not as a means for providing a positive pressure. Furthermore, HFNC is less expensive in terms of device cost and daily management than CPAP or BiPAP.

### 2.2. Mechanisms of Action

The mechanisms through which HFNC can improve respiratory functions are not precisely defined. However, results of some studies seem to indicate that HFNC can improve patient oxygenation acting on mechanisms strictly related to acute lung and/or diaphragm injury [15]. Heating and humidification of an inhaled gas do not only allow use of higher gas flows but favor secretion clearance and reduce inflammatory bronchoconstriction and respiratory workload. Moreover, clearance of CO2 from the anatomical dead space is facilitated and thoracic-abdominal coordination is improved [19]. Finally, a positive end respiratory pressure (PEEP) of about 2–6 cm H2O when gas flow is of 8–12 L/min in infants is generated [20]. In patients with acute hypoxemic failure, such as those with BR, this prevents collapse of the small airways during expiration, favors alveolar recruiting and reduces risk of pulmonary oedema shifting lung water from the alveoli to the perivascular interstitial space [21]. Table 1 summarizes the physiologic mechanisms responsible for the beneficial effects of HFNC.

### 2.3. Use in Children with Bronchiolitis (BR)

**Figure 1:**
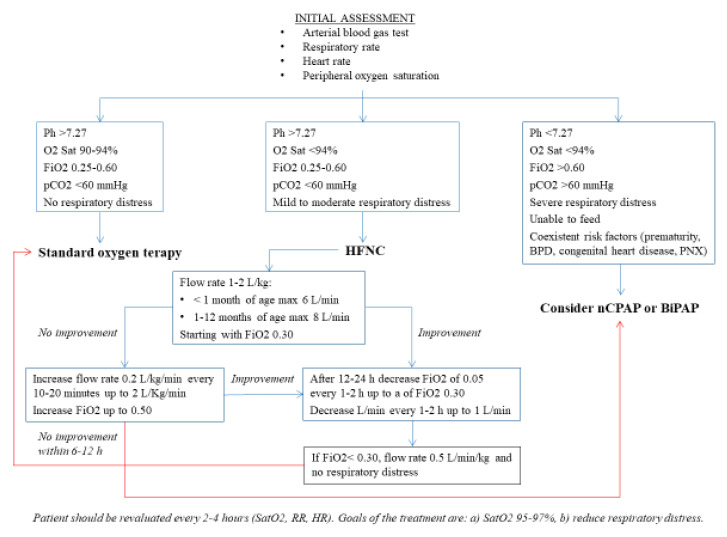
Practical flowchart about when to start standard oxygen therapy (SOT), high-flow nasal cannula (HFNC), or other methods of non-invasive ventilation in infants with bronchiolitis (BR). RR, respiratory rate; HR, heart rate.

Despite its large and increasing use in clinical practice, official guidelines for use of HFNC in children with respiratory failure are lacking. As for SOT, HFNC and other noninvasive oxygen delivery systems are recommended when saturation levels are persistently below 90–92% and should be suspended when saturation remains stable >94%, residual respiratory distress is minimal, and the child is feeding adequately. However, details on HFNC use are not precisely defined [9,10,11] and, frequently, decisions on how to conduct O2 therapy and the type of method to be used in the individual case is left to the opinion and experience of the operator. A recent survey across the USA including pediatric wards and pediatric intensive care units showed that only 37% had formal guidelines for HFNC initiation and 73% had weaning guidelines [22]. Generally, HFNC is not recommended in infants presenting with history of apneas, documented hemodynamic instability, pneumothorax, or craniofacial abnormalities, and should be used with caution in patients with a decreased level of consciousness, congenital heart disease, and chronic respiratory disease [23]. Regarding gas flow, in most of the studies the flow rate used varied from 2 to 10 L/min with variations mainly related to the intent to minimize patient’s breathing workload and oxygen saturation values. In some cases, flow rate was decided according to patient’s weight and generally varied between 1 and 2 L/kg/min [24,25,26,27,28,29]. Analysis of tolerance and efficacy of flow rates used in studies that have compared HFNC with other methods of oxygen administration seem to indicate that in infants with BR, flow rates of about 20 L/min or 2 L/kg/min can offer the highest efficacy without substantial risks of significant adverse events. In order to optimize results, FiO2 should be adjusted to achieve SaO2 of 95–97%, with the temperature 37 °C and relative humidity 100% [30,31,32,33]. Higher flow rates do not seem to improve efficacy and might induce reduced tolerance [34]. According to Mayfield et al. [35], markers of efficacy could be the decline of respiratory rate (RR) and heart rate (HR) of about 20% compared to the baseline values within the first 90 min of treatment. Increased risk of poor response has been found related to high hypercapnia levels and low increase in RR at baseline is associated with poor reduction of RR during treatment [36]. Regarding safety, HFNC is generally a safe procedure as in all the studies adverse events were only exceptionally described, generally in cases with inappropriate application of cannula size or an inappropriately high flow rate. In these cases, pneumothorax or air leak syndrome can occur [30,37]. Figure 1 summarizes a practical flowchart about when to start SOT, HFNC, or other methods of non-invasive ventilation in infants with BR.

## 3. Efficacy of High-Flow Nasal Cannula (HFNC) Oxygen Administration

Several studies have compared the efficacy and safety of HFNC to other measures of oxygen administration in order to evaluate when and how it could be used to optimize treatment of children with BR and respiratory failure.

### 3.1. HFNC vs. SOT

Most of the studies initially carried out to evaluate HFNC for BR treatment showed that this system of oxygen administration was safe and effective as it could reduce the risk of intubation and mechanical ventilation without important adverse events. However, the results of these studies must be interpreted with caution as in most of the cases they were not randomized controlled trials (RCTs), data were collected retrospectively, and methods used for analysis were debatable. Moreover, they did not allow the definitive establishment of whether HFNC was superior to SOT and could be used as a first measure to treat children with hypoxemic BR [35,38,39,40,41,42,43]. These questions remained unanswered even when only RCTs were considered. A systematic review and meta-analysis of four RCTs, including two studies carried out in China and published in Chinese with some methodological problems [30,44,45,46] showed that, compared to children treated with SOT, patients receiving HFNC oxygen administration had a similar length of hospital stay (LOS) (mean difference (MD) days −1.53, 95% CI −3.33 to 0.27, *p* = 0.10), length of oxygen supplementation (LOO) (MD days −0.59, 95% CI −1.70 to 0.53, *p* = 0.30), and incidence of intubation (relative risk (RR) 1.98, 95% CI 0.6 to 6.56, *p* = 0.26). Only the incidence of treatment failure was significantly lower in the HFNC group than in the SOT group (RR 0.50, 95%CI 0.40 to 0.62, *p* < 0.01). Despite this last finding, authors concluded [47] that HFNC did not significantly benefit children with BR compared with SOT and that further studies are needed to evaluate whether, when, and how HFNC has to be used to improve hypoxemic respiratory failure.

Slightly different conclusions can, however, be drawn from a more recent systematic review [48] in which data reported in one of the studies already included in the previous meta-analysis [34] and three different RCTs were pooled and evaluated [30,31,32,33]. A total of 1753 children under 2 years of age were studied. When all the children were considered together, most of the studied variables were similarly influenced by HFNC and SOT, as treatments did not differ in need for transfer to the pediatric intensive care unit, days of oxygen therapy, and LOS. Only the need for additional respiratory support, defined as intubation and mechanical ventilation, was significantly less frequent in the HFNC group than in controls (*p* < 0.001).

However, a more careful evaluation of the studies included in this systematic review seems to suggest that the comparable efficacy of the two oxygen delivery methods may depend on the characteristics of the children enrolled in the studies rather than on a real equivalence of their clinical efficacy. Most of the BR cases were very mild and the potential greater efficacy of HFNC in comparison to SOT for treatment of moderate and severe cases was not tested in an adequate number of patients. On the other hand, in the study in which both treatments were given to children admitted to the pediatric intensive care unit for moderate to severe disease, treatment failure rate, time to weaning off oxygen, length of pediatric intensive care unit stay, and LOS were all significantly lower in the HFNC group (*p* = 0.011, *p* < 0.001, *p* < 0.001, and *p* < 0.001, respectively) [33]. This explains why the authors concluded that, although there are insufficient data to support the use of HFNC for all children with hypoxemic BR, this method of oxygen supply seems the most appropriate rescue therapy for children who are not adequately supported by SOT.

### 3.2. HFNC vs. CPAP and BiPAP

Studies that have compared HFNC to CPAP/BiPAP for treatment of respiratory failure of children with BR have led to conflicting results and different suggestions for clinical use, although HFNC was generally associated with lower discomfort. This is clearly evidenced by the recent meta-analysis carried out by Moreel and Proesmans [48] in which three RCTs including a total of 213 children <24 months were pooled and analyzed. In two of these studies, those with the lowest number of enrolled patients [49,50], treatment failures were rare and similar in both groups of treatment whereas in the third study [51] treatment failure was significantly higher in the HFNC group. Details of the studies indicate that in all of them, HFNC was better tolerated than CPAP/BiPAP, as evidenced by the better CONFORT score in most of the children treated with HFNC. Regarding efficacy, in the trial by Sarkar et al. [49] only one patient in each group had no benefit from treatment and had to be intubated. Functional and subjective respiratory parameters such as RR, paO2, pCO2, and Respiratory Distress Assessment Index scores were similar at baseline and were similarly modified by the two treatments. In the study by Vahlkvist et al. [50], treatment failure was scarce in both groups. No significant differences in LOO or LOS, and in development of RR, pCO2, or Wood’s Clinical Asthma Score were observed. On the contrary, in the Milesi et al. study [51], where 342 young infants admitted to the PICU for moderate to severe BR were included, HFNC was found not equivalent to CPAP as failure occurred in 31.0% of the CPAP cases compared to 50.7%. Data were considered indicative of a relevant superiority of CPAP and a relative risk of success 1.63-times (95% CI 1.02–2.63) higher with CPAP compared to HFNC. Worsening of respiratory distress signs and discomfort were the leading cause of failure in the HFNC and CPAP groups, respectively. Global evaluation of these findings led the authors to conclude that there were insufficient data to support the use of HFNC therapy for all children with BR admitted to hospital because of hypoxemia and respiratory distress.

Conflicting results were also reported in other studies. Superiority of HFNC was evidenced by Clayton et al. [52] who evaluated the need for invasive mechanical ventilation in 6496 children with BR (median age 3.9 months) who were prescribed HFNC or CPAP/BiPAP as an initial respiratory treatment modality. Invasive mechanical ventilation was required in 12.3% of the cases and was more common in infants with CPAP/BiPAP than in those with HFNC (20.1% vs. 11.0%: *p* < 0.001). A need for intensive mechanical ventilation remained significantly higher even after adjustment for age, weight, race, viral etiology, presence of other comorbidities, and Pediatric Index of Mortality score (odds ratio, 1.53; 95% CI, 1.24–1.88). This led the authors to conclude that HFNC might be the preferred initial support modality for critically ill children with bronchiolitis.

Completely different results were, on the contrary, reported in other studies. Habra et al. [53] studied 137 children admitted to the pediatric intensive care unit: 77 were treated with HFNC, 10 with CPAP, and 50 with BiPAP. Failure rates of HFNC, defined as a change to another respiratory support modality or use of invasive mechanical ventilation, were significantly higher in the HFNC group compared with the group of children receiving CPAP or BiPAP (50.6% for HFNC vs. 0% for CPAP and 8% for BiPAP, *p* < 0.01). Among the 39 patients who failed HFNC, 90% were successfully shifted to BiPAP and weaned off later, whereas the other 4 required invasive mechanical ventilation. However, no differences among groups were evidenced in improvement of RR and HR after starting the intervention and during the first 48 h, and in terms of LOS or mortality. Authors stated that further studies are mandatory to evaluate the role of HFNC before this method can be preferred to CPAP or BiPAP to treat children with BR.

A similar conclusion is also expressed by the authors of a Danish study [54] that highlighted a greater effectiveness of CPAP in reducing RR and a need for oxygen, with a higher failure rate for HFNC; no difference emerged instead for LOO, LOS, and transfer to PICU. Table 2 [9,24,30,47,55,56] and Table 3 [34,48,52,54,57,58,59,60,61,62] summarize results from the main available studies. An overall evaluation of the results obtained in the studies that compared HFNC and CPAP/BiPAP indicates that at the moment it is not possible to establish whether, when, and in which subjects HFNC can replace CPAP/BiPAP in the treatment of bronchiolitis. To date, much of the difference between the studies can be ascribed to the diversity and inconsistent description of subjects’ characteristics and lack of uniformity in the way HFNC is used. Future studies should be better designed to avoid these shortfalls. Children with different degrees of respiratory failure were enrolled, different oxygen concentrations and flow were administered, and various criteria for failure definition were used. All these findings can lead to different results and inability to draw firm conclusions. The example of the study by Clayton et al. [52] in which HFNC was found superior to CPAP/BiPAP seems paradigmatic in this regard. The study was retrospective, and this cannot allow establishing causation. Moreover, it cannot be excluded that baseline clinical characteristics of children enrolled in the two groups were different and this could have influenced evolution of the disease. Markers of severe disease, such as hypoxemia, hypercarbia, dyspnea, encephalopathy, or apnea were not carefully collected in all the enrolled children. Finally, infants treated with CPAP/BiPAP were more severely ill than those given HFNC, as evidenced by the higher Pediatric Index Mortality 2 scores [62]. Moreover, the heterogeneity of the various healthcare settings where the studies were performed could have led to different outcomes in children receiving the same type of treatment. This is because the setting may influence the intensity of care provided, the familiarity with HFNC use, and the identification of treatment failure or clinical improvement.

## 4. Conclusions

Despite its widespread use, the real role of HFNC to treat respiratory failure in children with BR is not precisely defined. It is not established whether it can offer greater benefits compared to SOT, and when and in which infants it can substitute CPAP/BiPAP.

The evaluation of available study results suggests that children without severe respiratory impairment but persistent abnormal SaO2 despite SOT can be treated with HFNC with progressive higher flow in order to obtain normal SaO2. However, other respiratory support options like nCPAP or BiPAP must be used when respiratory distress is severe and response to HFNC is poor within the first hours of treatment.

## Figures and Tables

**Table 1:** Physiologic mechanisms responsible for the beneficial effects of high-flow nasal cannula (HFNC).

Effects of the High Flow	Effects of the Heated Gas	Effects of Controlled FiO2
Physiological dead space washout of waste gases including carbon dioxide (CO_2_)	Reduction in respiratory workload	No oxygen leak
Positive end-expiratory pressure	Reduction of bronchoconstriction	FiO2 up to 1.00 provided to the patient
Reduced airway resistance	Increased ciliary clearance	Better monitoring of oxygen saturation
Decreased respiratory rate	Better hydration of the mucosa	
Increased tidal volume	Better comfort	
Increased end-expiratory volume		

**Table 2:** Summary of retrospective studies and meta-analyses that compared HFNC vs. CPAP and BiPAP.

Authors	Year	N. Studies Evaluated	Disease, Type of Patients	Device	Outcome
Metge et al. [24]	July 2014	Retrospectively reviewed the medical records of all infants admitted to a pediatric intensive care unit at a tertiary care French hospital during the BR seasons of 2010/11 and 2011/12	Infants with acute BR	HFNC vs. nCPAP	HFNC is better tolerated, simpler, easier, and associated with less nasal trauma than CPAP. It needs to be confirmed whether HFNC should be used as a first approach in severe BR.
Heikkila et al. [55]	November 2018	Retrospective study	88 infants under 12 months with BR who received HFNC: 53 on paediatric wards and 35 in paediatric intensive care units	Treatment with HFNC	HFNC treatment was successful in 76 (86%) infants hospitalized in 53 pediatric wards and 23/35 ICU patients. In conclusion, HFNC is safe and often avoids the need for intensive care.
Lin et al. [47]	June 2019	Meta-analysis evaluating 9 RCTs	Infants with BR	Standard therapy with O2 vs. HFNC HFNC vs. nCPAP	This study suggests that HFNC was safe as initial respiratory treatment, but there is insufficient evidence to demonstrate benefits over SOT or nCPAP.
Luo et al. [56]	December 2019	A brief meta-analysis conducted on 8 studies	Infants and children with BR or pneumonia, with mild or severe hypoxemia	Standard therapy with O2 vs. HFNC HFNC vs. nCPAP	Among children <5 years of age with respiratory distress and mild hypoxemia, HFNC reduced the risk of treatment failure compared with SOT. However, nCPAP was associated with a lower risk of treatment failure than HFNC in children aged 1 to 6 months with severe respiratory distress and hypoxemia. No differences in intubation and mortality rates were found between HFNC and SOT or nCPAP.
Franklin et al. [30]	2019	9 studies	Children (<2 years) with acute BR	NCPAP vs. HFNC	The use of HFNC therapy reduced intubation rates.
Ralston [9]	July 2020	6 studies	Children with BR	Standard therapy with O2 vs. HFNC	No overall differences in the length of hospital stay or oxygen therapy between the groups.

**Table 3:** Summary of the clinical studies that compared HFNC vs. CPAP and BiPAP.

Authors	Year	Setting	Disease, Type of Patients	Respiratory Support, Device and Flow	Outcome
Milèsi et al. [34]	2017	A multicenter randomized controlled trial performed in 5 pediatric intensive care units	142 infants up to 6 months old with moderate to severe BR	nCPAP (7 cmH2O) vs. HFNC (2 L/kg/min)	nCPAP was slightly superior to HFNC in the initial respiratory support of these patients.
Pedersen et al. [54]	April 2017	A retrospective study between 2013 and 2015	49 children with BR	CPAP vs. HFNC	Evidence of greater effectiveness of CPAP in reducing RR and oxygen need with a higher failure rate for HFNC; no difference emerged, however, for the duration of treatment, the length of hospitalization, or transfer to pediatric intensive care unit.
Guillot et al. [57]	April 2018	Observational prospective study in a pediatric intensive care unit, during two consecutive seasons (2013–2014 without recommendation and 2014–2015 with a study design suggesting HFNC as first-line treatment)	Children with severe BR	HFNC vs. nCPAP HFNC vs. BiPAP	38% of children on HFNC therapy switched to nCPAP or BiPAP. A high pCO2 value has been correlated with a higher risk of HFNC therapy failure.
Clayton et al. [52]	February 2019	A retrospective study conducted in 92 American pediatric intensive care units	6496 children with BR	HFNC vs. nCPAP or BiPAP	nCPAP or BiPAP is associated with a greater risk of subsequent need for mechanical ventilation than HFNC. However, confounding factors such as the coexistence of disease or the use of sedatives in children with noninvasive positive pressure ventilation may have influenced the study results.
Suessman et al. [58]	December 2019	A retrospective study from January 2014–January 2018.	2657 children < 24 months of age with BR.	HFNC in PICU	Lower risk of intubation when heart rate decreased after HFNC application. Increased risk of intubation for infants less than 2 months of age, particularly on days 3 and 4 of RSV infection.
Nascimento et al. [59]	September 2020	A retrospective study from January 2016 to June 2017	81 children with BR in pediatric intensive care unit	HFNC in PICU	21% HFNC failure and need for non-invasive positive pressure ventilation or invasive ventilation.
Kamit et al. [60]	May 2020	A retrospective chart review between 1 January 2015 and 31 December 2016	84 patients with severe BR in pediatric intensive care unit	HFNC in PICU	The HFNC failure rate was 27.3%. Risk factors were significant tachycardia, dehydration, and a venous pH <7.30 at hospitalization.
Durand et al. [61]	July 2020	A multicenter RCT performed in 17 hospitals in Paris	268 infants aged <6 months with moderate BR	HFNC at 3L/kg over standard oxygen therapy	No difference was demonstrated in treatment failure (14% vs. 20%) or in pediatric intensive care unit admission risk (15% vs. 19%). Results were consistent with the review by Moreel et al. which do not support the preventive and routine use of HFNC in patients with moderate BR.
Moreel, Proesmans [48]	May 2020	Systematic literature search was performed in PubMed, Embase, and Cochrane Central Register of Controlled Trials (CENTRAL) from January 2000 to February 2020: pediatrics department and pediatric intensive care unit	Children (<2 years) with acute BR	4 studies HFNC vs. oxygen. 3 studies nCPAP vs. HFNC	HFNC seems more appropriate for children who are not adequately supported by oxygen, but there are insufficient data to support the use of HFNC therapy for all children with BR.
Vahlkvist et al. [50]	March 2020	Randomized clinical trial	50 children with BR	HFNC vs. nCPAP	CPAP and HFNC are comparable in terms of treatment duration, treatment failure, and hospital stay, and have similar effects on respiratory rate, pCO2, and oxygen supply requirement.

## Data Availability

Not applicable.
